# Recent Progresses in Electrochemical DNA Biosensors for SARS-CoV-2 Detection

**DOI:** 10.3389/fbioe.2022.952510

**Published:** 2022-07-15

**Authors:** Yanqiu Mei, Xiaofeng Lin, Chen He, Weijia Zeng, Yan Luo, Chenghao Liu, Zhehao Liu, Min Yang, Ying Kuang, Qitong Huang

**Affiliations:** ^1^ Key Laboratory of Prevention and Treatment of Cardiovascular and Cerebrovascular Diseases, Ministry of Education, Key Laboratory of Biomaterials and Biofabrication in Tissue Engineering of Jiangxi Province, Key Laboratory of Biomedical Sensors of Ganzhou, School of Public Health and Health Management, School of Medical and Information Engineering, Gannan Medical University, Ganzhou, China; ^2^ Oil-Tea in Medical Health Care and Functional Product Development Engineering Research Center in Jiangxi, The Science Research Center, School of Pharmacy, Gannan Medical University, Ganzhou, China

**Keywords:** COVID-19, SARS-CoV-2, electrochemical DNA biosensors, nucleic acid detection, virus/protein detection

## Abstract

Coronavirus disease 19 (COVID-19) is still a major public health concern in many nations today. COVID-19 transmission is now controlled mostly through early discovery, isolation, and therapy. Because of the severe acute respiratory syndrome coronavirus 2 (SARS-CoV-2) is the contributing factor to COVID-19, establishing timely, sensitive, accurate, simple, and budget detection technologies for the SARS-CoV-2 is urgent for epidemic prevention. Recently, several electrochemical DNA biosensors have been developed for the rapid monitoring and detection of SARS-CoV-2. This mini-review examines the latest improvements in the detection of SARS-COV-2 utilizing electrochemical DNA biosensors. Meanwhile, this mini-review summarizes the problems faced by the existing assays and puts an outlook on future trends in the development of new assays for SARS-CoV-2, to provide researchers with a borrowing role in the generation of different assays.

## Introduction

The severe acute respiratory syndrome coronavirus 2 (SARS-CoV-2), a new member of the β-coronavirus genus, is closely related to SARS-CoV and is also the seventh coronavirus to infect humans (Akalin et al., 2020; Wiersinga et al., 2020; Zu et al., 2020), causing severe respiratory symptoms such as fever (37.3°C), cough and expectoration, nasal obstruction or even dyspnea in humans. A few people developed gastrointestinal symptoms (GI) (Jin et al., 2020a), such as nausea, vomiting, and diarrhea. Although the 2003 SARS-CoV, 2012 Middle East Respiratory Syndrome (MERS-COV), and the current epidemic of SARS-CoV-2 are all Human coronaviruse (HCoV) strains, SARS-CoV-2 is more infectious and pathogenic. At present, the main sources of infection of the disease include patients with COVID-19 patients, asymptomatic infections, and latent infections, which can be transmitted from person to person through droplets, contact, aerosols, and other transmission routes (Anderson et al., 2020; Kalbusch et al., 2020; Lin et al., 2020; Fu et al., 2021; Huang et al., 2021; Pan et al., 2021; Yip et al., 2021). It has been reported that one COVID-19 patient can transmit to three people at the same time (Qing et al., 2020), and the population is generally susceptible, especially elderly patients with chronic diseases who are more likely to become critically ill (Adhikari et al., 2020).

**FIGURE 1:**
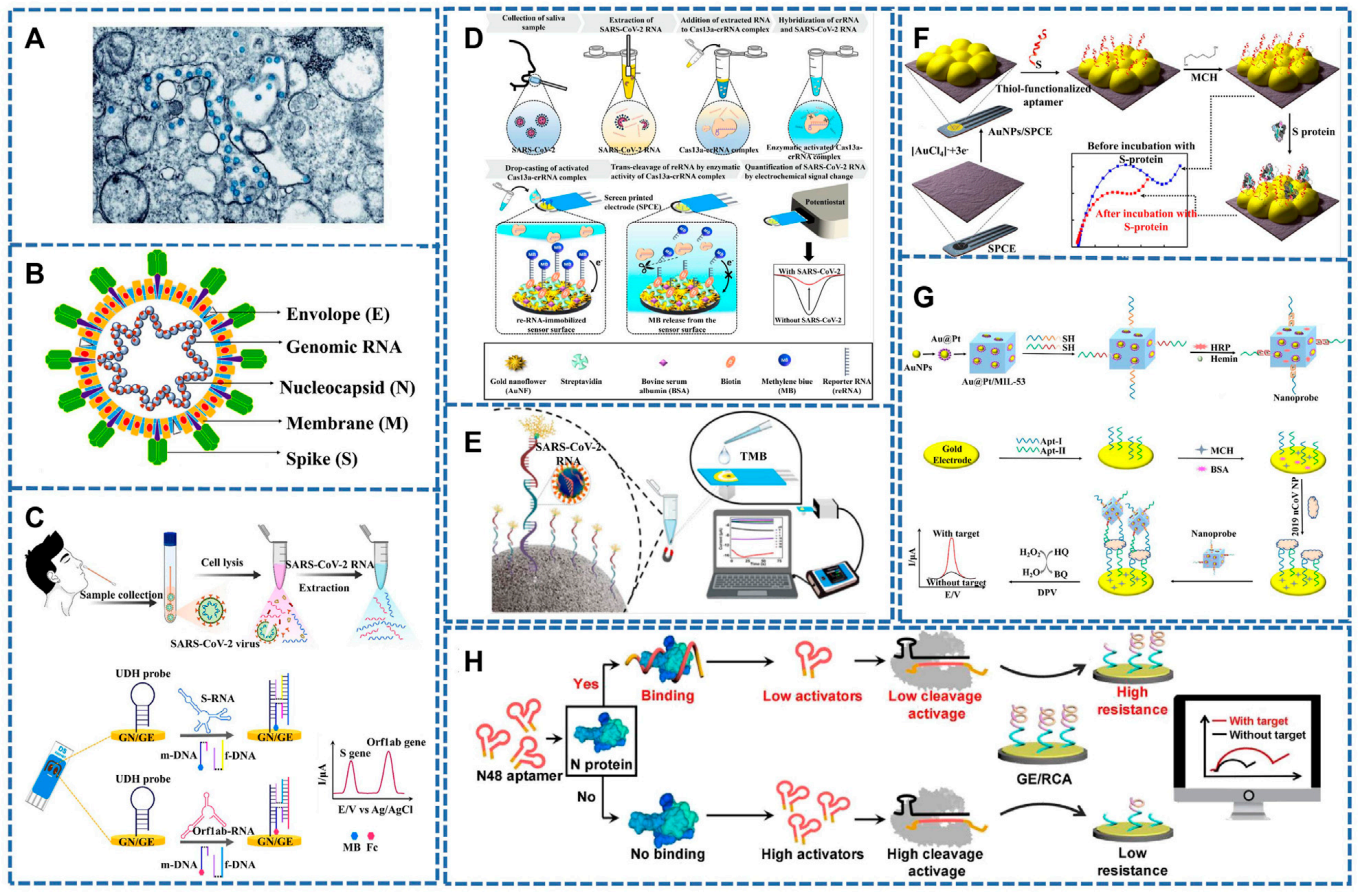
**(A)** TEM image of SARS-CoV-2 (the blue globules) (Bullock and Tamin, 2020). **(B)** SARS-CoV-2’s typical structure, S, M, E, and N proteins, encapsulates genomic RNA within virus particles (Kirtipal et al., 2020). **(C)** A detection workflow of SARS-CoV-2 RNA sequences from clinical samples using the electrochemical biosensor for detection of the S and Orf1ab genes (Kashefi-Kheyrabadi et al., 2022). **(D)** Schematic diagram of an electrochemical biosensing strategy for SARS-CoV-2 detection using CRISPR/Cas13a. (Heo et al., 2022). **(E)** Schematic diagram of an electrochemical gene sensor for detecting SARS-COV-2 (Cajigas et al., 2022). **(F)** Diagram of the preparation steps of the sensor for detecting SARS-CoV-2 S protein. (Abrego-Martinez et al., 2022). **(G)** Schematic diagram of constructing electrochemical sensor based on Au@Pt/MIL-53 (Tian et al., 2021). **(H)** Strategy and workflow of SARS-COV-2 antigen detection by CRISPR-based electrochemical sensor (Liu et al., 2022).

The single-stranded positive-stranded RNA virus, SARS-CoV-2, is 80–220 nm in diameter and has 12–24 nm vesicular rod like spikes. (Figure 1A) (Bullock and Tamin, 2020; Huang et al., 2020c; Lu et al., 2020; Zhou et al., 2020). At the same time, it is also a single-stranded positive-stranded RNA virus with typical “coronavirus” morphological characteristics. Its homology with SARS-COV and MERS genome sequences is close to 79% and 50%, respectively (Lu et al., 2020; Zhou et al., 2020), so it can be modified to detect SARS-CoV-2 by referring to previous detection methods of SARS-COV. The genome of SARS-CoV-2 consists of two noncoding (5′-terminal noncoding region and 3′-terminal noncoding region) and five coding regions (an open reading box 1a/b (ORF1a/b), the S region encoding spinous glycoprotein (S protein), the E region encoding envelope protein (E protein), the M region encoding membrane protein (M protein) and the N region encoding nucleocapsid protein (N protein)) (Xu et al., 2020; Yang and Wang, 2020) (Figure 1B). Among them, ORF1a/b genes are responsible for viral genome replication, transcription and translation (Jalandra et al., 2020; Kirtipal et al., 2020; Udugama et al., 2020; Yang and Wang, 2020). The virus on the surface of the outer coating is mainly composed of four structural proteins, the S protein, the E protein, the M protein and the N protein (Nie et al., 2020). S protein, which enables the virus to enter the host cell, can be used for vaccine, research and development of therapeutic antibodies and diagnosis depending on its advantages. Proteins M and E are responsible for forming the envelope of the virus, whereas the N protein participates in the assembly of viruses (Jalandra et al., 2020; Udugama et al., 2020; Yang and Wang, 2020).

At the early stage of the outbreak, metagenomic sequencing obtained the whole genome sequence of the SARS-CoV-2 virus, which provided the basis for the classification of SARS-CoV-2 as a new member of the genus β-coronavirus. Although metagenomic sequencing is costly, it not only provides technical support for the analysis of the origin and pathogenesis of SARS-CoV-2 but also lays the foundation for the development of novel SARS-CoV-2 detection methods (Lu et al., 2020; Wu et al., 2020). As detection methods have improved, the commonly used methods include polymerase chain reaction (PCR) (Yuan et al., 2020), reverse transcription PCR (RT-PCR) (Huang et al., 2020a; Hu et al., 2020; Xie et al., 2020), reverse transcription loop-mediated isothermal amplification (RT-LAMP) (Baek et al., 2020; Yan et al., 2020), regularly interspaced short palindromic repeats-CRISPR-associated (CRISPR-Cas) (Broughton et al., 2020; Ding et al., 2020) and nucleic acid biosensors (Alafeef et al., 2020; Zhu et al., 2020). Currently, Although PCR can yield results with less viral genetic material. However, we still need more sensitive, simple, accurate, and inexpensive rapid assays for SARS-CoV-2 detection.

Electrochemical biosensors could provide the possibility for rapid medical diagnosis by rapid detection of biomarkers (Chen et al., 2018; Huang et al., 2020b; Lu et al., 2021; Raza et al., 2021; Huang et al., 2022), which have been used to detect Zika virus (Afsahi et al., 2018), Ebola virus (Ilkhani and Farhad, 2018), HIV(Nandi et al., 2020), influenza virus (Krishna et al., 2016; Chowdhury et al., 2019), etc. With the advancement of COVID-19 research, electrochemical biosensors, especially electrochemical DNA biosensors, have been widely studied in the detection of SARS-CoV-2.

This mini-review covers the recent progress of the rapid detection of SARS-COV-2 by using electrochemical DNA biosensors for the first time. What’s more, this mini-review summarizes the problems faced by the existing assays and puts an outlook on future trends in the development of new assays for SARS-CoV-2, which can provide researchers with a borrowing role in the development of new assays.

## Electrochemical DNA Sensor Detection of SARS-COV-2

Electrochemical DNA biosensors for the detection of viruses and bacteria are now widely used due to their advantages of higher sensitivity, less sample size, low cost, simplicity, and portability (Adam et al., 2020; Lin et al., 2021; Huang et al., 2022; Mei et al., 2022). The electrochemical DNA biosensors are based on the single-strand DNA or complementary target DNA fixed on the electrode and the target DNA through the principle of base complementarity, resulting in changes in concentration, energy, and other aspects, and this change can be converted into visual electrical signals through the appropriate conversion elements on the sensor (Huang et al., 2020b; Karimi-Maleh et al., 2021). Therefore, they combine biological specific recognition with electrochemical high sensitivity analysis to further realize the detection and analysis of the target, so that it has the potential of immediate detection and diagnosis.

### Specific Detection of SARS-COV-2 Nucleic Acid

Recently, nanomaterials have been extensively applied in life science, energy science and other fields (Xu et al., 2019; Zhuang et al., 2019; Kuang et al., 2020; Wen et al., 2020; Chen et al., 2021; He et al., 2021; Liu et al., 2021; Savchenko et al., 2021; Schultz et al., 2021; Wu et al., 2021; Xu and Liu, 2021; He et al., 2022; Yi et al., 2022; Zhuang et al., 2022). At present, nano-materials in electrochemical biosensors have also been broadly concerned for gauging SARS-COV-2. Graphene (G) is considered one of the suitable materials for sensor applications due to its special good properties such as physical properties, electronics as well as oxygen-containing groups, which are introduced in the construction of biosensors and can improve the analytical parameters of electrochemical biosensors (Abdel-Haleem et al., 2021; Meng et al., 2021). Alafeef et al. developed an electrochemical biosensor chip constructed by G that can selectively recognize SARS-CoV-2 RNA (N gene) (Alafeef et al., 2020). It was obtained by immobilizing highly specific ssDNA (antisense oligonucleotides)-capped gold nanoparticles (AuNPs) probes of viral N gene onto G coated filter paper. AuNPs achieve the goal of improving the electrochemical response of the sensor to the target by enhancing the electron transfer process and providing a large surface area for the ssDNA probe. G-ssDNA-AuNPs-built monitoring platform can read the results in 5 min with a handheld reader. When combined with benchtop devices, point-of-care (POC) testing can be offered to economically lag, resource-poor areas. Zhao et al. 2021) synthesized an ultra-sensitive sandwich electrochemical sensor using calixarene functionalized go and SARS-CoV-2 targeted RNA with limit of detection (LOD) as low as 200 copies/mL for clinical samples. Based on this sensor, a smartphone can detect SARS-CoV-2. Moreover, the detection process can detect SARS-CoV-2 samples sensitively, accurately and rapidly without RNA amplification, which also provides effective suggestions for low-cost and simple POC diagnosis.

Rolling circle amplification (RCA) and catalytic hairpin assembly (CHA) in the nucleic acid amplification strategy can be combined with electrochemical analysis to accurately amplify electrochemical signals. Chaibun et al. (2021) designed the electrochemical biosensor for SARS-CoV-2 (S or N gene) RNA based on RCA. The technique can detect viruses as low as one copy/μl in 2 h by differential pulse voltammetry (DPV). It was evaluated in conjunction with quantitative RT-PCR (qRT-PCR) on 106 clinical samples, of which 41 were positive (SARS-COV-2) and nine other samples were positive for other respiratory viruses, with 100% agreement between the two methods. Peng et al. (2021) prepared an electrochemical sensor based on CHA and terminal deoxynucleotidyl transferase (TdT) induced polymerization. When the target gene (ORF1ab gene) combined with hairpin HP1 and HP2 to form a Y-type DNA structure, TdT induced polymerization was further activated. A great quantity of long single-stranded DNA products are generated in the dNTP pool, during which a large number of Ru(NH_3_)_6_^3+^ adsorbs on the DNA phosphoric acid skeleton through strong electrostatic interaction. Therefore, this method can be used to obtain evidently boosted electrochemical signals for sensitive monitoring of SARS-COV-2. Kashefi-kheyrabadi et al. (2022) developed an electrochemical sensor without nucleic acid amplification. The detection process is shown in Figure 1C, the S and ORF1ab genes of SARS-CoV-2 can be detected simultaneously within 1 h with LOD as low as 5.0 and 6.8 ag/μl.

Heo et al. (2022) combined CRISPR/Cas13a with the electrochemical biosensor. The detection process is shown in Figure 1D. When the single-stranded RNA (ssRNA) probe was recognized by the SARS-CoV-2 RNA phase, the Cas13a-crRNA complex was formed, which was then introduced into the reporter RNA (reRNA)-coupled electrochemical sensor to activate RNase, thereby cutting reRNA. In this process, redox molecules released by reRNA will cause changes in their current, thus achieving the purpose of sensitive detection. Amplification-free sensors designed by the team enable ultra-low concentration testing of SARS-CoV-2 RNA. This opens the possibility of on-site and high-speed diagnostic COVID-19 testing.

Although many sensors are available to monitor SARS-COV-2, false-positive results from its homologous viruses cannot be ruled out. Cajigas et al. (2022) reported an electrochemical biosensor that can specifically detect SARS-COV-2 and distinguish the homologous viruses of SARS-CoV, Middle East Respiratory Syndrome (MERS), and Human Coronavirus (HKU1) (Figure 1E). Biosensors with immobilized capture probes were combined with modified magnetic beads (MMBS), while the capture probes were first hybridized with the targets and then hybridized with biotinylated signal probes in a sandwich format. The biotinylated signal probe allowed interaction with one of three distinct protein-enzyme compounds containing distinct numbers of horse radish peroxidase (HRP) molecules to generate visual electrical signals by the timing current method. The prepared biosensor provides the possibility to check infected and asymptomatic patients. At the same time, it will also contribute to resisting the COVID-19 pandemic.

### Specific Detection of SARS-COV-2 VIRUS/PROTEIN

With the development of nucleic acid testing, there are broad prospects for virus detection, but if the viral RNA is mutated, it can produce false-negative results, so researchers switched to detecting SARS-CoV-2-related proteins. Such as receptor-binding domain (RBD) (Jalandra et al., 2020; Udugama et al., 2020), S protein (Jin et al., 2020b; Wu et al., 2020) and N protein. Therefore, Abrego-Martinez et al. (2022) prepared a biosensor for detecting SARS-CoV-2 (S protein) by fixing probe ssDNA on gold nanoparticles (Figure 1F). Its advantages were fast detection speed, low detection limit (1.30 p.m.), and results that could be obtained in 40 min, but SARS-CoV had a certain response to it, which was negligible compared to SARS-CoV-2. The direct detection of SARS-CoV-2 was achieved by immobilizing ssDNA AuNPs on screen-printed electrode, which was also applied in a handheld potentiostat linked to a smartphone. To further reduce costs, Curti et al. prepared a biosensor targeting SARS-CoV-2 S1 by using an inexpensive and highly conductive single-wall carbon nanotube screen-printed electrode (SWCNT-SPE). The adapted ssDNA can block virus infection *in vitro*. It is possible to prepare multifunctional sensors (Curti et al., 2022).

Designing a highly sensitive assay is urgent for the early diagnosis and treatment of SARS-CoV-2. Tian et al. (2021) designed a diaptamer sensor for highly selective recognition of SARS-CoV-2 N protein using the metal-organic framework MIL-53 Au@Pt Nanoparticles and enzymes, as shown in the Figure 1G. The detection limit was as low as 8.33 pg/ml.

CRISPR-Cas is an efficient, simple and powerful gene targeting technology (Liu and Fan, 2014), and the nucleic acid detection of CRISPR/Cas nuclease holds great promise for the development of SARS-COV-2 diagnostics under high sensitivity, specificity and reliability (Chertow Daniel, 2018; Li et al., 2019). Liu et al. (2022) combined this method with electrochemical analysis to prepare a sensor for SARS-COV-2 virus detection (Figure 1H). the signal of electrochemical impedance spectroscopy (EIS) signal was linked to the morphology and presence of the RCA-DNA structure, which was determined by the DNA cleavage activity of Cas12a attachment regulated by target-induced competition. Therefore the sensor was also responsible for its ability to test SARS-COV-2 with high specificity.

The nucleic acid and virus/protein determinations are summarised in Table 1. Compared to the traditional testing methods (PCR, RT-PCR), electrochemical DNA sensors have demonstrated their ability to detect SARS-CoV-2 with ease of use, no need for expensive instruments, lower detection limits, higher sensitivity, and specificity. In particular, it has the advantage of shorter detection times, demonstrating its ability to detect and control outbreaks rapidly. The miniaturised electrochemical biosensor can be combined with lateral flow assay (LFA), loop-mediated isothermal amplification (LAMP), RT-LAMP approaches, clustered regularly interspaced short palindromic repeats (CRISPR) and other methods to further increase sensitivity. In fact, we also hope that by comparing the different assays, other researchers will be able to create more sensitive, rapid, economical, and accurate COVID-19 assays.

**TABLE 1:** Comparison of SARS-CoV-2 electrochemical genetic sensor detection methods.

Targets for detection	Methods	Linear range	LOD	References
S protein	EIS	0–10^5^ pM	1.30 pM	Abrego-Martinez et al. (2022)
N gene	Microcontrollers	585.4–5.854 × 10^7^ copies/μl	6.9 copies/μl	Alafeef et al. (2020)
RBD protein	EIS	10–6.4 × 10^4^ nM	7 nM	Amouzadeh Tabrizi and Acedo, (2022)
RdRP gene	DPV	10^−10^–10^−5^ M	1.86 × 10^−7^ M	Ang et al. (2022)
N gene	CV	800–4,000 copies/µl	258.01 copies/µl	Avelino et al. (2021)
SARS-CoV-2 RNA (H)	I-t	0–1,000 pM	0.73 pM	Cajigas et al. (2022)
N or S gene	DPV	1–10^9^ copies/μl	1 copies/µl	Chaibun et al. (2021)
N gene	EIS	0.1–10^6^ fg/ml	0.59 fg/ml	Cui et al. (2022)
S1 protein	DPV	0.3–300 nM	7 nM	Curti et al. (2022)
ORF1ab gene	DPV	10^2^–10^9^ fg/ml	100 fg/ml	Damiati et al. (2021)
ORF1a gene	CV	—	2.3 copies/µl	Najjar et al. (2021)
RdRP gene	DPV	100–3 × 10^6^ fM	45 fM	Deng et al. (2022)
RdRP gene	ECL	1–10^5^ fM	2.67 fM	Fan et al. (2021)
RdRP gene	ECL	10–10^7^ aM	7.8 aM	Fan et al. (2022)
RdRP gene	CV	1–8 × 10^3^ pM	0.3 pM	Farzin et al. (2021)
ORF1ab gene	ECL	50–10^8^ fM	0.514 fM	Gutiérrez-Gálvez et al. (2022)
N protein	DPV	50–10^5^ pg/ml	16.5 pg/ml	Han et al. (2022)
ORF1ab gene	DPV	1–10^9^ aM	0.48 aM	Hatamluyi et al. (2022)
ORF and S genes	DPV	1.0 × 10^−1^–1.0 × 10^5^ fg/ml	ORF gene: 4.4 × 10^−2^ fg/ml. S gene: 8.1 × 10^−2^ fg/ml	Heo et al. (2022)
S protein	SWV	10^−4^–10^2^ nM	10 nM	Idili et al. (2021)
ORF1ab gene	ECL	0.1–10^11^ fM	0.1 fM	Jiang et al. (2022)
ORF1ab and S genes	SWV	10^−16^–10^−11^ M	ORF1ab gene: 5.0 ag/μl. S gene: 6.8 ag/μl	Kashefi-Kheyrabadi et al. (2022)
RdRP and N genes	DPV	10^3^–10^9^ copies	RdRP gene: 0.972 fg/μl. N gene: 3.925 fg/μl,	Kim et al. (2021)
N gene	DPV	10–200 pg/μl	10 pg/μl	Kumar et al. (2021)
N protein	EIS	0.05–125 ng/ml	0.077 ng/ml	Liu et al. (2022)
ORF1ab gene	DPV	0–100 pM	1.01 pM	Martínez-Periñán et al. (2021)
SARS-CoV-2 RNA	Chronoamperometric	1–10^4^ pM	1 pM	Pang et al. (2021)
ORF1ab gene	DPV	10^2^–10^6^ pM	26 fM	Peng et al. (2021)
ORF1ab and N genes	SWV	10^−3^–10 ng/μl	3.8 × 10^−5^ ng/μl	Ramírez-Chavarría et al. (2022)
S protein	DPV	10–50 ng/ml	2.63 ng/ml	Sari et al. (2022)
N gene	DPV	10–10^6^ fM	3.5 fM	Song et al. (2021)
N protein	DPV	25–5 × 10^4^ pg/ml	8.33 pg/ml	Tian et al. (2021)
RdRP gene	ECL	1–10^5^ fM	0.21 fM	Yao et al. (2021)
RdRp gene	ECL	0–2000 aM	43.70 aM	Zhang et al. (2022a)
RdRP gene	ECL	0–1,000 aM	32.8 aM	Zhang et al. (2022b)
RdRP gene	ECL	0–10^3^ aM	12.8 aM	Zhang et al. (2022c)
RdRP gene	ECL	0–3,000 aM	59 aM	Zhang et al. (2022d)
ORF1ab gene	DPV	10^3^–10^9^ copies/ml	200 copies/ml	Zhao et al. (2021)

Abbreviation: CV, Cyclic voltammetry; DPV, Differential pulse voltammery; EIS, Electrochemical impedance spectroscopy; SWV, Square wave voltammetry; ECL, Electrochemiluminescence; I-t, Amperometric.

## Conclusion and Perspectives

The rapid, sensitive and accurate determination of SARS-CoV-2 is crucial for the prevention and control of the epidemic. Electrochemical DNA biosensors have the advantages of high sensitivity, high selectivity, and economical portability, et al., which have been gradually applied to SARS-CoV-2 detection. In this mini-review, the latest researches on electrochemical DNA biosensors for the monitoring of SARS-CoV-2 in recent years have been summarized. Although there are many electrochemical DNA biosensors for SARS-CoV-2 detection, there are still some opportunities and challenges: 1) the preparation of working electrode materials should be simpler; 2) the stability of the electrochemical DNA biosensors should be guaranteed; 3) how to achieve simultaneous detection of multiple different genes? Of course, with the further in-depth research, the above problems will be effectively solved, which will provide the possibility for the commercial application of electrochemical biosensors for SARS-CoV-2 detection.
